# Physicochemical Properties of Starch Isolated from Betahealth, a High β-Glucan Barley Cultivar

**DOI:** 10.3390/foods14183226

**Published:** 2025-09-17

**Authors:** Jin-Cheon Park, Gyeong A Jeong, Seul-Gi Park, Young-Mi Yoon, On-Sook Hur, Chang Joo Lee

**Affiliations:** 1 Department of Crop Science, National Institute of Crop and Food Science, Rural Development Administration, Wanju 55365, Jeonbuk, Republic of Korea; jinchun5214@korea.kr (J.-C.P.); ahsia1004@korea.kr (S.-G.P.); mi3710@korea.kr (Y.-M.Y.); oshur09@korea.kr (O.-S.H.); 2 Department of Food Science and Biotechnology, Wonkwang University, Iksan 54538, Jeonbuk, Republic of Korea; jka0719@naver.com

**Keywords:** barley starch, β-glucan, Betahealth, barley cultivar

## Abstract

This study investigated the physicochemical properties of starch from the newly developed β-glucan-rich barley cultivar Betahealth. The cultivar was bred through a three-way cross between Betaone (F1, Shikoku Hadaka 97 × Glacier AC38) and Dahyang, and its potential as a high-β-glucan food supplement was evaluated. Betahealth’s general composition comprised 11.8%, 1.06%, 2.74%, 3.66%, 56.6%, and 12.3% protein, ash, crude fat, amylose, starch, and β-glucan, respectively. The compositional characteristics of the parent cultivars varied among developed cultivars. The average starch granule size decreased in the following order: Dahyang (12.2 μm), Shikoku Hadaka 97 (11.4 μm), Glacier AC38 (8.63 μm), and Betahealth (6.96 μm). Granule size greatly influenced gelatinization properties, with smaller granules showing higher onset, peak, and conclusion temperatures during gelatinization. Gelatinization temperatures significantly differed among samples, except in Betahealth. Amylose content strongly correlated with pasting properties, with Shikoku Hadaka 97 (10.4%) and Betahealth (8.75%) showing lower amylose content than Glacier AC38 (43.4%) and Dahyang (43.8%). Thus, differences in starch granule size, gelatinization properties, and pasting characteristics depended on cultivar, suggesting that these factors are important for selecting cultivars suitable for specific processing applications.

## 1. Introduction

Barley (*Hordeum vulgare* L.) is among the world’s oldest cultivated cereals and currently the fifth most extensively produced crop, following wheat, corn, rice, and soybeans [1]. Renowned for its high nutritional value and abundance of bioactive constituents such as dietary fiber, barley has increasingly attracted global interest for diverse applications in the food and industrial sectors [2,3,4]. In recent years, considerable attention has been directed toward breeding and processing strategies aimed at modifying the starch structure of barley to enhance its quality characteristics and physiological functionality [5,6,7]. β-Glucan, a major functional component in barley, is a water-soluble polysaccharide composed of (1→3)(1→4)-β-D-glucan linkages and is primarily present in endosperm cell walls. β-Glucan exhibits physiological activities such as lowering blood cholesterol, regulating blood glucose levels, and improving gut microbiota balance, and its health functionality has been recognized by organizations including the European Food Safety Authority (EFSA) [2,8]. Recent studies have reported that the molecular weight and solubility of β-glucan determine the viscosity in the gastrointestinal tract, which directly contributes to the reduction in LDL cholesterol and glycemic response [3,4,9]. Bai et al. reported that thermal processing significantly affects the molecular characteristics and antioxidant properties of β-glucan in highland barley [9]. Islam et al. reported that germination treatment markedly enhances the physicochemical characteristics and bioactive properties of Betaone, a barley cultivar rich in β-glucan [10]. Tang et al. further reported that the structural distribution of barley β-glucan exerts a profound effect on digestibility and colonic fermentation, thereby reinforcing its role as a physiologically functional dietary fiber [11]. These results indicate that β-glucan provides various health benefits, including lowering LDL cholesterol, suppressing postprandial blood glucose levels, improving gut microbiota balance, and reducing oxidative stress. To maximize these functional traits, a new barley cultivar Betaone was developed by crossbreeding the Canadian cultivar Glacier AC38, known for its excellent starch quality and yield, with the Japanese cultivar Shikoku Hadaka 97 (high β-glucan content and desirable grain characteristics) [10,12]. Betaone combines high β-glucan content, easy threshing, and favorable starch properties, suggesting its potential as a functional food ingredient [10,13]. Betaone exhibits dual health benefits in animal studies, including blood glucose regulation and fatty liver improvement [13,14]. Barley starch accounts for approximately 65–70% of the grain and is increasing valued as a staple food ingredient owing to its antioxidant activity, glycemic index regulation, and cholesterol-lowering effects [15]. As a major component in various foods, starch exhibits cultivar-dependent physicochemical differences that directly influence food processing and product development [16]. Therefore, studying starch properties is essential, as they affect the physical properties and processing suitability of food ingredients [15]. This study aimed to investigate the physicochemical characteristics of starch from Betahealth*,* a newly developed barley cultivar with elevated β-glucan content, to evaluate its potential for application as a food ingredient.

## 2. Materials and Methods

### 2.1. Materials

Four barley cultivars (Shikoku Hadaka 97, Glacier AC38, Dahyang, and Betahealth), cultivated in 2022 at the National Institute of Crop Science following the Rural Development Administration’s standard cultivation practices [17], were used in this study. The cultivars were selected based on the genetic background of Betahealth, which has a high β-glucan content. Betahealth is a newly developed cultivar bred by crossing the F1 hybrid Betaone (Shikoku Hadaka 97 × Glacier AC38) as the female parent with the Korean cultivar Dahyang as the male parent. In this study, Betahealth and its parental lines were selected as comparison groups. Harvested grains were dried to <13% moisture content and subsequently polished using a laboratory-scale rice mill (TM-05, Satake Co., Hiroshima, Japan). Polished samples were ground using a cyclone sample mill equipped with a 0.5 mm screen (ZM-300, Retsch GmbH, Haan, Germany) for further analysis.

### 2.2. Barley Starch Isolation

Starch was isolated from cultivated barley using a modified method based on Andersson et al. [18] and Bae et al. [19], involving NaOH treatment. Fifty grams of ground barley flour were mixed with 30 mL of distilled water to form a dough. The dough was subsequently blended with 500 mL of 0.2% NaOH for 5 min, followed by soaking for 24 h. A 0.2% NaOH solution was used, as this concentration minimizes structural alterations of starch granules and does not cause significant changes in their crystalline structure and crystallinity [18]. To compensate for the low concentration, the samples were immersed for 24 h to ensure the effective removal of non-starch impurities. After steeping, the supernatant was discarded, and the precipitate was washed five times with distilled water. The washed samples were centrifuged at 1500× *g* for 20 min to remove the supernatant. After adding distilled water, the mixture was filtered through a 53 μm metal sieve and centrifuged again at 1500× *g* for 20 min to remove the supernatant. The precipitate was further mixed with 250 mL of distilled water for 5 min and centrifuged at 1500× *g* for 20 min to remove brown impurities. The resulting starch was washed with ethanol and dried at 40 °C until the moisture content was <10%. The dried starch was ground and passed through a 75 μm sieve (ASTM 200, CISA, Barcelona, Spain) for analysis.

### 2.3. Composition of Barley Grains and Barley Starch

The compositional analysis of barley was performed according to AOAC methods [20]. Protein content was determined using an elemental analyzer (Vario MAX N, Elementar Analysensysteme GmbH, Hanau, Germany) by measuring nitrogen content and applying a conversion factor of 6.25 according to AOAC 979.09. Crude fat was analyzed using the Soxhlet extraction method (Soxhlet System HT 1043 extraction unit Foss Tecator, Höganäs, Sweden) with ether according to AOAC 930.09. Ash content was determined by direct ashing using a muffle furnace (DS-DMF-14, LAB24, Japan) at 550 °C for 8 h according to AOAC 923.03. Amylose content was analyzed using a commercial kit (Megazyme Ltd., Bray, Ireland). The total β-glucan content of both barley and isolated starch was measured using the McCleary method [21] with a β-glucan assay kit (Megazyme Ltd.) and a UV/Vis spectrophotometer (U-2800, Hitachi Instruments Inc., Tokyo, Japan) at an absorbance of 510 nm. Total starch content was determined using the Megazyme kit (Megazyme Ltd.) following the AACC method [22].

### 2.4. Shape and Particle Size of Barley Starch

Barley starch granule morphology was observed using a scanning electron microscope (Phenom Pro-X, Phenom-World, Eindhoven, The Netherlands). Starch samples were mounted on carbon tape, coated with gold, and examined under vacuum conditions at an accelerating voltage of 10 kV and a magnification of ×1000. Particle size distribution was measured using a laser diffraction particle size analyzer (LS 13 320, Beckman Coulter, Inc., Fullerton, CA, USA) with ethanol as the dispersant, and the average granule size of barley grains and separated barley starch was determined [11].

### 2.5. X-Ray Diffraction of Barley Starch

X-ray diffraction patterns of barley starch were analyzed using an X-ray diffractometer (MiniFlex-600, Rigaku, Tokyo, Japan) according to the method described by Na et al. [23]. The analysis was conducted under the following conditions: diffraction angle (2*θ*) range of 3–30°, target: Cu-Kα, filter: Cu-Kα, voltage: 40 kV, current: 15 mA, and step time: 2 s.

### 2.6. Gelatinization Properties of Barley Starch

Gelatinization properties of isolated barley starch were analyzed using a differential scanning calorimeter (DSC 4000, Perkin-Elmer, Waltham, MA, USA). Starch and distilled water were placed in a DSC pan at a ratio of 10 mg starch:40 mg water and equilibrated at room temperature for 2 h. Thereafter, samples were heated from 30 °C to 130 °C at a rate of 5 °C/min. From the resulting endothermic curves, the onset (*T_o_*), peak (*T_p_*), conclusion temperatures (*T_c_*), and gelatinization enthalpy (Δ*H*: crystal melting enthalpy) were determined.

### 2.7. Pasting Properties of Barley Starch

Pasting properties of barley starch were measured using a rapid visco analyzer (RVA-4500, Perten Instruments, Hägersten, Sweden). A 3 g starch sample (dry basis, adjusted to 14% moisture content) was mixed with distilled water in an aluminum canister. The measurement was conducted over 13 min following the AACC method [22]. Pasting temperature, final viscosity, breakdown, and setback values were obtained from the resulting pasting curve.

### 2.8. Statistical Analysis

All experimental data were conducted in triplicate and analyzed using SPSS (version 23.0; SPSS Inc., Chicago, IL, USA). An analysis of variance (ANOVA) was performed, followed by Duncan’s multiple range test. Values are expressed as the mean ± standard deviation, and statistical significance was defined as *p* < 0.05.

## 3. Results and Discussion

### 3.1. Composition of Barley Grains and Barley Starch

Table 1 presents the compositional analysis of barley and isolated barley starch. Betaone, developed by crossing Shikoku Hadaka 97 and Glacier AC38, contained 11.3% β-glucan [24]. Further crossing with the Korean cultivar Dahyang produced Betahealth, which exhibited an increased β-glucan content of 12.3% (Table 1). These results indicated that Betahealth is a newly bred cultivar with enhanced functional properties, particularly owing to its increased β-glucan content, known for its physiological effects such as reducing blood cholesterol levels and regulating blood glucose. β-Glucan, a major functional component of barley, is typically present at 3.4% to 7.1% [14]. As a water-soluble dietary fiber located in the barley cell wall, β-glucan lowers blood cholesterol levels, contributing to cardiovascular disease prevention [8]. Betahealth’s composition was 11.8% protein, 1.06% ash, 2.74% crude fat, 3.66% amylose, 56.6% starch, and 12.3% β-glucan. The compositional profiles of Shikoku Hadaka 97, Glacier AC38, and Dahyang also varied depending on cultivar (Table 1). Barley generally contains approximately 70% starch, 5–10% β-glucan, 2–3% crude fat, and 5–12% protein. However, these compositional characteristics vary depending on genetic background and cultivation environment [25,26].

The amylose content of Betahealth was notably lower than that of its parental lines, which is associated with variations at the waxy (Wx-1) locus regulating amylose synthesis [27]. The granule-bound starch synthase I (GBSSI) enzyme expressed at the Wx-1 locus is the key enzyme responsible for amylose biosynthesis, and previous studies have reported that waxy or partial-waxy lines exhibit reduced GBSSI activity, resulting in lower amylose content [28]. Such a reduction in amylose content may lead to alterations in the gelatinization and retrogradation properties of starch, serving as an important factor influencing the starch characteristics of Betahealth. The total starch content of barley starch isolated from barley flour was 97.1% in Shikoku Hadaka 97, 92.1% in Glacier AC38, 92.6% in Dahyang, and 97.3% in Betahealth. Shikoku Hadaka 97 (10.4%) and Betahealth (8.75%) exhibited waxy amylose characteristics, whereas Glacier AC38 (43.4%) and Dahyang (43.8%) displayed non-waxy types. According to Andersson et al. [18], protein, ash, and β-glucan contents in barley starch differ by starch type (nonwaxy, high amylose, and waxy) and cultivar.

### 3.2. Particle Morphology and Size Distribution of Barley Starch

Table 2 shows the particle size distributions of domestically cultivated barley and their isolated starches. Barley flour showed an average particle size of 237–263 μm, and no significant differences were detected among the cultivars (*p* > 0.05). This result is considered to be due to the presence of various non-starch components such as proteins, fibers, cell walls, and residues, which led to a relatively larger particle size compared with isolated starch. In contrast, the average particle sizes of starch isolated from dehulled barley flour were 12.2 μm for Dahyang, 11.4 μm for Shikoku Hadaka 97, 8.63 μm for Glacier AC38, and 6.96 μm for Betahealth, with Betahealth having the smallest starch granules. Barley starch is composed of A-type granules, (diameters of approximately 15–25 μm) and smaller, spherical B-type granules (diameter ~10 μm), which are spherical in shape [16]. Shikoku Hadaka 97 (d_50_ 12.1 μm) and Dahyang (d_50_ 12.7 μm) contained a higher proportion of A-type granules, whereas Glacier AC38 (d_50_ 8.35 μm) and Betahealth (d_50_ 7.02 μm) showed a greater distribution of the smaller B-type granules. In general, A-type starch granules are larger and more crystalline, exhibiting greater resistance to digestive enzymes, whereas B-type granules are smaller with a larger surface area and are more readily hydrolyzed by enzymes [15,16,29]. Therefore, differences in starch granule distribution are a key factor directly related to resistant starch formation, regulation of glycemic response, and processing suitability. In this study, all cultivars exhibited a mixture of A- and B-type starch granules, with noticeable variations in granule size distribution depending on the cultivar. Starch particle size distribution varied by cultivar, and such structural differences significantly influence the starch’s functional properties, processing suitability, gelatinization behavior, and digestibility [6].

**Figure 1:**
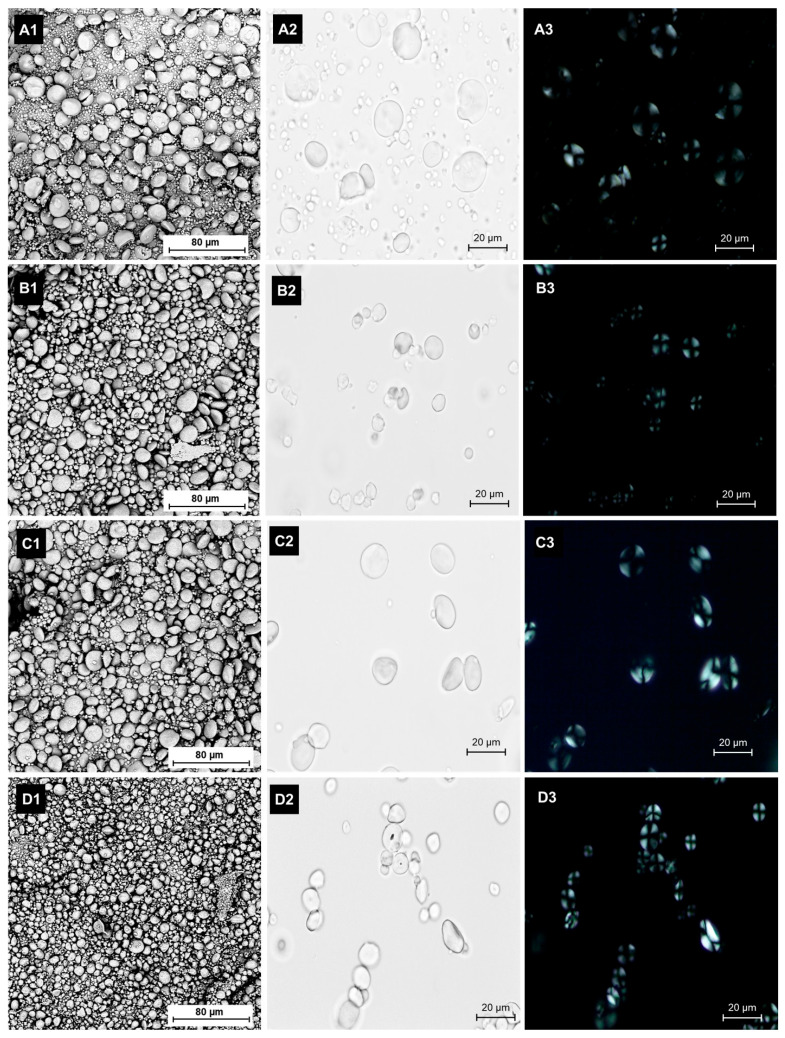
Morphology of barley starch granules under (1) scanning electron microscopy (2) light microscopy, and (3) polarized light. (**A**) Shikoku Hadaka 97, (**B**) Glacier AC38, (**C**) Dahyang, and (**D**) Betahealth.

The morphology of barley starch granules is shown in Figure 1. Barley starch granules exhibited a round to oval shape, with granule sizes varying by cultivar. This observation aligns with findings of Bae et al. [19]. Betahealth starch granules primarily consisted of small-sized particles, with an average size of 6.96 μm, followed by Glacier AC38 (8.63 μm), Shikoku Hadaka 97 (11.4 μm), and Dahyang (12.2 μm). Residual materials such as proteins and fibers were removed during starch isolation, and polarized light microscopy confirmed intact granules without particle damage via the presence of Maltese crosses [24,30]. Variations in particle size and distribution are expected to affect starch physicochemical properties, including gelatinization and viscosity.

### 3.3. X-Ray Diffraction Patterns of Barly Starch

**Figure 2:**
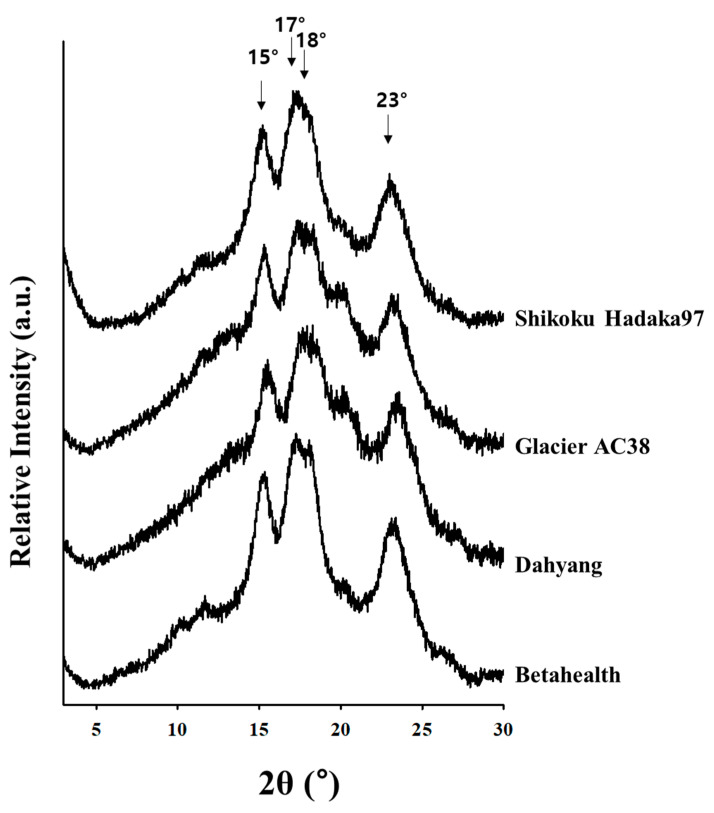
X-ray diffraction patterns of barley starches: Shikoku Hadaka 97, Glacier AC38, Dahyang, and Betahealth.

The X-ray diffraction patterns of starches from four barley cultivars are shown in Figure 2. All barley starch samples exhibited distinct diffraction peaks at approximately 2*θ* values of 15°, 17°, 18°, and 23°, indicating a typical A-type starch crystalline structure [31]. Zhu [15] reported that barley starch exhibits an A-type polymorph regardless of granule size, supporting the present findings. Starches obtained from mutants deficient in ssIIa, ssIIa (in *amo1* mutant), and ΔSBEIIa/ΔSBEIIb (with reduced expression levels of both ΔSBEIIa and ΔSBEIIb) were found to display a B-type polymorph together with a V-type polymorph, accompanied by reduced crystallinity. In contrast, starches from the ΔSBEIIa and ΔSBEIIb mutants exhibited an A-type polymorph. These changes in polymorphic type may be attributed to alterations in starch composition and the molecular structures of both amylose and amylopectin. In particular, longer chains and increased intermediate chain length of amylopectin tend to increase the occurrence of a B-type polymorph.

### 3.4. Gelatinization Parameters of Barley Starch

Table 3 presents the gelatinization properties of barley starch. The onset (*T_o_*), peak (*T_p_*), and conclusion temperatures (*T_c_*) of barley starches showed increased as the average particle size decreased, with Betahealth having the smallest average particle size (6.96 μm), followed by Glacier AC38 (8.63 μm), Shikoku Hadaka 97 (11.4 μm), and Dahyang (12.2 μm). Significant differences were not observed among cultivars, except for Betahealth (*p* < 0.05). Structural differences, such as starch particle size and amylose content, are closely associated with the functional and physical properties of starch [32]. Among these factors, particle size has a notable influence on gelatinization properties, with smaller granules showing higher onset, peak, and conclusion temperatures [29,31]. In addition, cultivars with higher amylose content, such as Dahyang and *Glacier AC38*, require more energy to disrupt double-helical associations, resulting in greater crystalline stability. In contrast, Shikoku Hadaka 97 and Betahealth, which have relatively lower amylose contents, exhibited lower Δ*H* values. This observation is consistent with previous studies reporting that amylose molecules promote double-helix formation and reinforce crystalline lamellae, thereby increasing gelatinization enthalpy [29,33]. A similar trend was observed in this study, where *T_o_*, *T_p_*, and *T_c_* showed negative correlations with the average particle size (r = −0.779, r = −0.787, r = −0.721, respectively, *p* < 0.01). Gelatinization enthalpy (Δ*H*) represents the energy required to disrupt and completely dissolve the double-helical structures of starch molecules [30]. Cultivar Δ*H* values were 7.7 J/g for Shikoku Hadaka 97, 8.2 J/g for Glacier AC38, 8.7 J/g for Dahyang, and 7.5 J/g for Betahealth, with no significant differences observed among cultivars (*p* > 0.05). Betahealth exhibited overall higher gelatinization temperatures, attributed to its higher proportion of small starch granules with B-type characteristics than other cultivars. According to Stevnebø et al. [34], starch digestibility varies depending on granule size, and smaller starch granules may contribute to a lower hydrolysis rate and reduced postprandial glycemic response than larger granules. Particle size differences among cultivars resulted in variations in gelatinization properties, which may serve as an important criterion for selecting barley cultivars according to their processing suitability for specific applications [11].

### 3.5. Pasting Properties of Barley Starch

**Figure 3:**
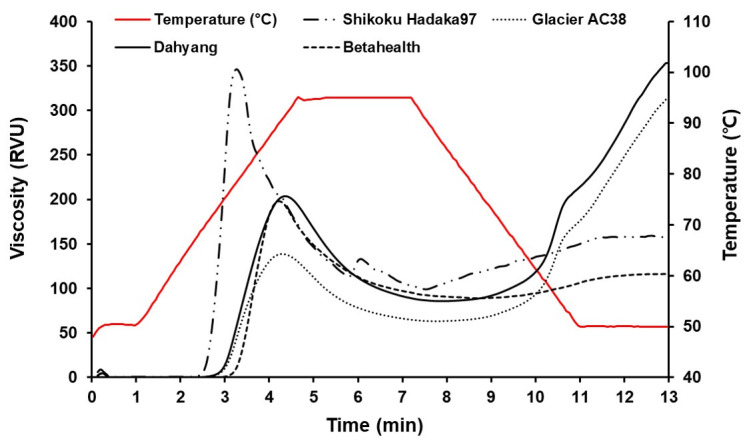
Pasting properties of barley starches measured using a rapid visco analyzer: Shikoku Hadaka 97, Glacier AC38, Dahyang, and Betahealth.

The pasting properties of barley starch are shown in Figure 3. The waxy barley cultivar Shikoku Hadaka 97 exhibited a peak viscosity of 346.25 RVU, trough of 98.92 RVU, breakdown of 247.33 RVU, final viscosity of 157.5 RVU, and setback of 58.58 RVU, indicating low final viscosity and trough values compared to peak viscosity. Betahealth, another waxy barley cultivar, showed a similar trend with a peak viscosity of 197.52 RVU, final viscosity of 116.17 RVU, and setback of 27.25 RVU. Barley starches are classified as waxy or nonwaxy types, and waxy exhibit significantly lower final viscosity and setback values relative to peak viscosity [35]. These characteristics are attributed to the low amylose and high amylopectin content in waxy barley starches. Rapid visco analyzer (RVA) provides a rapid, quantitative assessment of starch pasting properties, and the viscosity profile enables indirect evaluation of functional properties such as amylose content, gel-forming ability, and starch degradation resistance [5]. In contrast to waxy cultivars, nonwaxy barley starches with high amylose content, such as Glacier AC38 (43.4%) and Dahyang (43.8%), exhibited higher final viscosities (312.42 RVU and 352.92 RVU, respectively) and setback (248.92 RVU and 267 RVU) relative to their peak viscosity (139.08 RVU and 203.5 RVU, respectively). As amylose content increased, final viscosity (r = 0.985, *p* < 0.01) and setback (r = 0.996, *p* < 0.01) also increased, whereas peak viscosity (r = –0.633, *p* < 0.01), trough (r = –0.729, *p* < 0.01), and breakdown (r = –0.594, *p* < 0.01) showed decreased. Therefore, amylose content significantly correlated with RVA pasting properties and played a key role in starch thermal stability and retrogradation tendency [36]. Barley starch’s physical properties and processing suitability appeared significantly influenced by factors such as starch purity, granule size, and the content and ratio of amylose and amylopectin.

## 4. Conclusions

This study investigated the physicochemical properties of Betahealth, a newly developed barley cultivar with enhanced β-glucan content, produced through the crossbreeding of Betaone (F1, Shikoku Hadaka 97 × Glacier AC38) and Dahyang. Betahealth’s composition included 11.8% protein, 1.06% ash, 2.74% fat, 3.66% amylose, 56.6% starch, and 12.3% β-glucan. The parental cultivars, Shikoku Hadaka 97, Glacier AC38, and Dahyang, showed cultivar-dependent compositional differences. The isolated starch granules consisted of A-type granules (large and lenticular) and B-type granules (smaller and more spherical). The average starch granule size decreased in the order: Dahyang (12.2 μm) > Shikoku Hadaka 97 (11.4 μm) > Glacier AC38 (8.63 μm) > Betahealth (6.96 μm). Starch granule size significantly influenced gelatinization properties. Smaller granules showed higher onset, peak, and conclusion temperatures, although significant differences were only observed in Betahealth. In pasting properties, higher amylose content was associated with increased final viscosity and setback, whereas peak viscosity, trough, and breakdown decreased. Amylose content played a critical role in determining starch thermal stability and retrogradation tendency. Barley cultivars differed in starch granule size, gelatinization, and pasting properties, important factors in determining processing suitability and cultivar selection for specific applications.

## Figures and Tables

**Table 1:** Chemical compositions of barley grain and starches.

Sample	Varieties	Composition (%) ^1^						
		Moisture	Ash	Protein	Total Starch	β-Glucan	Lipids	Amylose
Whole flour	Shikoku Hadaka 97	10.1 ± 0.01 ^b^	0.82 ± 0.01 ^b^	11.2 ± 0.09 ^b^	68.7 ± 0.35 ^b^	5.81 ± 0.00 ^c^	1.11 ± 0.01 ^c^	6.56 ± 0.00 ^c^
	Glacier AC38	9.42 ± 0.06 ^c^	0.80 ± 0.02 ^b^	10.7 ± 0.20 ^c^	68.3 ± 0.18 ^b^	8.79 ± 0.00 ^b^	1.45 ± 0.04 ^b^	31.1 ± 0.16 ^a^
	Dahyang	10.3 ± 0.04 ^a^	0.59 ± 0.00 ^c^	10.9 ± 0.23 ^bc^	73.6 ± 0.16 ^a^	5.30 ± 0.02 ^d^	1.03 ± 0.02 ^d^	21.6 ± 0.56 ^b^
	Betahealth	9.31 ± 0.04 ^c^	1.06 ± 0.02 ^a^	11.8 ± 0.12 ^a^	56.6 ± 0.45 ^c^	12.3 ± 0.04 ^a^	2.74 ± 0.02 ^a^	3.66 ± 0.20 ^d^
Starch	Shikoku Hadaka 97	6.46 ± 0.02 ^b^	0.17 ± 0.01 ^c^	0.27 ± 0.01 ^a^	97.1 ± 0.07 ^a^	0.06 ± 0.01 ^a^	0.13 ± 0.04 ^c^	10.4 ± 0.02 ^b^
	Glacier AC38	6.46 ± 0.03 ^b^	0.21 ± 0.01 ^b^	0.16 ± 0.01 ^b^	92.1 ± 0.18 ^b^	0.01 ± 0.00 ^b^	0.21 ± 0.01 ^ab^	43.4 ± 0.12 ^a^
	Dahyang	6.46 ± 0.16 ^b^	018 ± 0.02 ^c^	0.15 ± 0.04 ^b^	92.6 ± 0.64 ^b^	0.01 ± 0.00 ^b^	0.24 ± 0.04 ^a^	43.8 ± 0.51 ^a^
	Betahealth	7.92 ± 0.02 ^a^	0.34 ± 0.01 ^a^	0.19 ± 0.02 ^b^	97.3 ± 0.35 ^a^	0.01 ± 0.01 ^b^	0.17 ± 0.01 ^bc^	8.75 ± 0.02 ^c^

^1 a–d^ The values with different superscripts within a column are significantly different by Duncan’s multiple range test (*p* < 0.05).

**Table 2:** Granule size of barley grain and starches.

Sample	Varieties	GSD (Granule Size Distribution) ^1^				
		Mean (μm) ^2^	Median (μm)	D_10_ (μm)	D_50_ (μm)	D_90_ (μm)
Whole flour	Shikoku Hadaka 97	237 ± 5.55 ^a^	212 ± 6.95 ^a^	8.44 ± 0.38 ^a^	212 ± 6.95 ^a^	526 ± 5.75 ^a^
	Glacier AC38	259 ± 14.1 ^a^	237 ± 15.7 ^a^	8.75 ± 0.16 ^a^	237 ± 15.7 ^a^	549 ± 13.3 ^a^
	Dahyang	238 ± 27.4 ^a^	212 ± 34.3 ^a^	9.49 ± 0.65 ^a^	242 ± 34.3 ^a^	513 ± 39.8 ^a^
	Betahealth	263 ± 37.8 ^a^	232 ± 33.6 ^a^	7.83 ± 1.66 ^a^	232 ± 33.6 ^a^	592 ± 87.7 ^a^
Starch	Shikoku Hadaka 97	11.4 ± 0.05 ^b^	12.1 ± 0.08 ^b^	1.63 ± 0.03 ^b^	12.1 ± 0.08 ^b^	20.0 ± 0.05 ^a^
	Glacier AC38	8.63 ± 0.05 ^c^	8.35 ± 0.05 ^c^	2.59 ± 0.53 ^a^	8.35 ± 0.05 ^c^	14.8 ± 0.08 ^b^
	Dahyang	12.2 ± 0.07 ^a^	12.7 ± 0.08 ^a^	1.72 ± 0.01 ^b^	12.7 ± 0.08 ^a^	19.9 ± 0.03 ^a^
	Betahealth	6.96 ± 0.05 ^d^	7.01 ± 0.03 ^d^	1.09 ± 0.01 ^c^	7.02 ± 0.05 ^d^	11.9 ± 0.13 ^c^

^1^ d_10_, d_50_, and d_90_: granule size at which 10%, 50%, and 90% of all the granules by volume are smaller. ^2 a–d^ The values with different superscripts within a column are significantly different by Duncan’s multiple range test (*p* < 0.05).

**Table 3:** Gelatinization parameters of barley starches.

Varieties	Gelatinization Parameters ^2^				
	*T_o_* ^1^ (°C)	*T_p_* (°C)	*T_c_* (°C)	*T_c_*–*T_o_* (°C)	Δ*H* (J/g)
Shikoku Hadaka 97	61.4 ± 0.24 ^a^	68.0 ± 0.29 ^a^	82.3 ± 1.25 ^b^	20.9 ± 1.45 ^ab^	7.7 ± 0.44 ^a^
Glacier AC38	62.0 ± 0.36 ^a^	68.2 ± 0.17 ^a^	80.1 ± 1.13 ^ab^	18.2 ± 1.48 ^ab^	8.2 ± 1.09 ^a^
Dahyang	61.8 ± 0.17 ^a^	68.0 ± 0.12 ^a^	79.3 ± 1.07 ^a^	17.5 ± 1.09 ^a^	8.7 ± 0.38 ^a^
Betahealth	68.5 ± 1.36 ^b^	77.7 ± 0.57 ^b^	90.1 ± 0.96 ^c^	21.6 ± 2.27 ^b^	7.5 ± 1.15 ^a^

^1^ *T_o_*: onset temperature; *T_p_*: peak temperature; *T_c_*: conclusion temperature; Δ*H*: gelatinization enthalpy. ^2 a,b,c^ The values with different superscripts within a column are significantly different by Duncan’s multiple range test (*p* < 0.05).

## Data Availability

The original contributions presented in the study are included in the article, further inquiries can be directed to the corresponding author.

## Abbreviations

The following abbreviations are used in this manuscript:

GSD	granule size distribution
*T_o_*	onset temperature
*T_p_*	peak temperature
*T_c_*	conclusion temperature
Δ*H*	gelatinization enthalpy
RVA	rapid visco analyzer
